# Data mining-based discriminant analysis as a tool for the study of egg quality in native hen breeds

**DOI:** 10.1038/s41598-022-20111-z

**Published:** 2022-09-23

**Authors:** Antonio González Ariza, Ander Arando Arbulu, Francisco Javier Navas González, José Manuel León Jurado, Juan Vicente Delgado Bermejo, María Esperanza Camacho Vallejo

**Affiliations:** 1 Department of Genetics, Faculty of Veterinary Sciences, University of Córdoba, 14071 Córdoba, Spain; 2 Animal Breeding Consulting S.L., 14014 Córdoba, Spain; 3 Andalusian Institute of Agricultural and Fisheries Research and Training (IFAPA), Alameda del Obispo, 14004 Córdoba, Spain; 4 Agropecuary Provincial Centre, Diputación of Córdoba, 14071 Córdoba, Spain

**Keywords:** Animal breeding, Data mining, Data processing, Statistical methods

## Abstract

Despite the wide biodiversity of avian species of zootechnical interest in Spain, projects aimed at characterizing these genotypes and their products are necessary. External and internal egg quality traits were measured in 819 eggs laid by hens of 10 different genotypes: White, Franciscan, Black and Partridge varieties of Utrerana, Blue Andalusian, Spanish White-Faced, Andalusian Tufted White and Black varieties, Araucana; and Leghorn Lohmann LSL-Classic lineage (commercial hybrid line) hen breeds. After multicollinearity analysis of egg quality-related traits was performed (VIF ≤ 4), major diameter, minor diameter, egg weight, and albumen height were deemed redundant explanatory variables and discarded. A stepwise discriminant canonical analysis was developed to cluster eggs across hen genotypes considering egg quality attributes. Shell a* and b* variables reported the highest discriminant power (Wilks’ lambda: 0.699 and 0.729, respectively). The first two discriminant functions captured 60.48% of the variance across groups (F1: 39.36%; F2: 21.12%). Clear quality differentiation signs are evidenced for Mediterranean native breeds’ eggs when compared to Leghorn’s eggs. Consequently, this evidence of egg quality differentiation may favor the standardization of breed- and variety-linked distinctive products, which may open new market opportunities based on the existence of a wide spectrum of diet or culinary applications.

## Introduction

In recent years, consumers have shown increasing interest in animal products that are obtained through sustainable production systems. The purpose of sustainable systems is to obtain differentiated food with a low impact on the environment and human health and to consider animal welfare^1^. Most of the eggs consumed worldwide are laid by hens from commercial hybrid lines^2^. However, a new market niche is emerging for products with special characteristics closely linked to native breeds and traditional breeding systems^3^. As a result, the existence of local breeds may eventually lead to the parallel development of alternative production systems and the fixation of populations in rural areas, which in turn may contribute to the prevention of biodiversity loss and the disappearance of animal genetic resources^4^.

The acceptability of specific products by consumers has been reported to depend on quality traits related to the eggshell, albumen, and yolk^5^. Depending on the need to break the egg to measure quality features, these can be classified into external or internal quality traits^6^. Previous research reported that the quality of hen eggs can be influenced by genetic and nongenetic components, such as the age of the hen, feed intake and environmental and meteorological factors^7–9^. Egg parameters have been reported to influence fertility, embryo development, hatchability, and chicken viability^10^.

Spanish Atlantic and Mediterranean trunks cluster together all the hen breeds that spread across the territory. The hens of the Atlantic trunk are generally semiheavy birds, with red earlobes and brown-shelled eggs. On the other hand, the Mediterranean population comprises light individuals, with white earlobes and white-shelled eggs^11^. Egg production under alternative poultry systems promotes and sets its basis on the use of local hen breeds, which are able to efficiently produce differentiated products under adverse weather conditions^12^. Contextually, Andalusia (southern Spain) is influenced by the Mediterranean climate, with very high temperatures from May to October; hence, only certain autochthonous laying hen genotypes (Utrerana, Blue Andalusian, Spanish White-Faced and Andalusian Tufted) are adapted enough to thrive when kept in the traditional backyard and extensive conditions of the area^6^.

Several studies have focused on disentangling the existing genetic, productive and reproductive differences within the varieties of Utrerana avian breeds and across Andalusian autochthonous breeds^5–7,13,14^. In this regard, discriminant canonical analysis approaches have been suggested as a validation tool for Utrerana egg commercial quality classification depending on internal and external quality-related traits^15^.

Contextually, González Ariza et al.^15^ designed a tool comprising six discriminant functions which were able to significantly determine whether specific eggs may correctly fit the features of the different commercial size categories (S, M, L, and XL), across the Utrerana hen breed varieties. In this manner, the tool evidenced eggs from different varieties may fit different niche opportunities as they may cover particular sections of the market for egg consumption.

To this aim, the present study seeks to determine the differential clustering patterns of egg quality-related traits from the eggs laid by four Spanish native breeds (white-shelled egg layers) and their varieties: Utrerana (Franciscan, White, Black and Partridge), Blue Andalusian, Spanish White-Faced and Andalusian Tufted breeds (White and Black) in comparison to Araucana breed as a foreign native breed outgroup (American continent) and a control flock of a commercial laying lineage. The outcomes of the present study may support the characterization and typification of the entity of the products derived from Spanish laying breeds as a strategy to plan potential marketing and commercialization alternatives to support the sustainability of the breeding program of those endangered genotypes.

## Results

### Descriptive statistics

The mean, standard deviation, maximum, minimum and percentiles for each egg quality-related trait of the study are shown in Supplementary Table S1.

### Canonical discriminant analysis model reliability and explanatory potential

**Figure 1:**
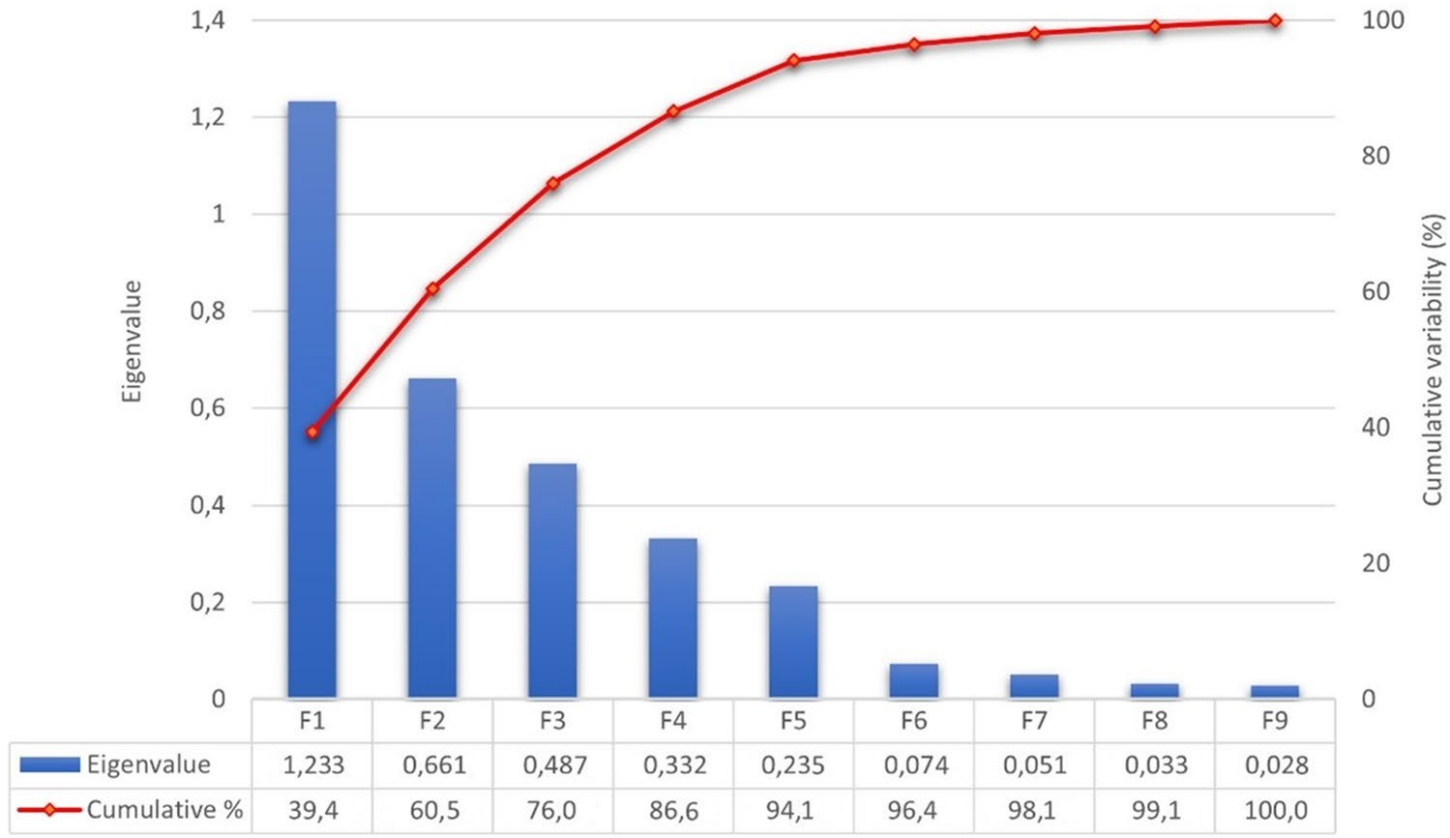
Canonical variable functions and percentages of self-explained and cumulative variance.

Major diameter, minor diameter, egg weight, and albumen height were discarded from the analyses because they presented VIF values over 4 (Table 1). Significant Pillai´s trace criterion (Value: 1.8923; df1: 180; df2: 7173; P < 0.0001) determined that discriminant canonical analysis was feasible. As reported in Table 2, out of the nine discriminant functions designed after the analyses, seven presented a significant discriminant ability. The discriminatory power of the F1 function was high (eigenvalue of 1.23; Fig. 1), with 60.48% of the variance being explained by F1 and F2.

**Table 1:** Multicollinearity analysis of quality-related traits of eggs.

Statistics/parameters	Tolerance (1 − R^2^)	VIF
Yolk weight	0.46	2.18
Shell b*	0.46	2.18
Shell L*	0.48	2.08
Yolk diameter	0.48	2.07
Eggshell weight	0.53	1.88
Eggshell strength	0.54	1.86
Yolk color fan	0.58	1.71
Resistance area	0.59	1.70
Albumen weight	0.67	1.50
Yolk a*	0.69	1.45
Eggshell thickness	0.69	1.45
Yolk L*	0.74	1.35
Yolk b*	0.78	1.28
Albumen pH	0.80	1.24
Haugh units	0.81	1.23
Shell a*	0.83	1.20
Shape index	0.86	1.16
Yolk pH	0.89	1.13
Visual defects	0.96	1.04

Interpretation thumb rule: VIF = 1 (not correlated); 1 < VIF < 4 (moderately correlated); VIF ≥ 4 (highly correlated).

**Table 2:** Canonical discriminant analysis efficiency parameters to determine the significance of each canonical discriminant function.

Test of function(s)	Wilks' lambda	Chi-square	df	Sig
1 through 9	0.082	1214.592	171	< 0.001
2 through 9	0.199	786.456	144	< 0.001
3 through 9	0.381	468.933	119	< 0.001
4 through 9	0.583	262.279	96	< 0.001
5 through 9	0.734	150.466	75	< 0.001
6 through 9	0.817	98.074	56	< 0.001
7 through 9	0.884	60.241	39	0.016
8 through 9	0.942	29.213	24	0.212
9	0.981	9.242	11	0.600

*df* degrees of freedom.

### Canonical coefficients, loading interpretation and spatial representation

Variables were ranked depending on their discriminating properties. For this, a test of equality of group means across egg quality classification was used (Table 3). Lower values of Wilks’ lambda and greater values of F indicate a better discriminating power, which translates into a better position in the rank. The analyses revealed that yolk and white pH did not significantly contribute (P < 0.05) to the discriminant ability of significant discriminant functions.

**Table 3:** Results for the tests of equality of group means to test for difference in the means across egg groups once redundant variables have been removed.

Variable	Wilks’ lambda	F	df1	df2	*p*-value	Rank
Shell a*	0.70	38.63	9	808	< 0.0001	1
Shell b*	0.73	33.44	9	808	< 0.0001	2
Albumen weight	0.77	27.16	9	808	< 0.0001	3
Shape index	0.81	20.77	9	808	< 0.0001	4
Haugh units	0.82	19.74	9	808	< 0.0001	5
Yolk weight	0.86	14.82	9	808	< 0.0001	6
Eggshell weight	0.86	14.76	9	808	< 0.0001	7
Yolk diameter	0.88	12.25	9	808	< 0.0001	8
Shell L*	0.88	12.10	9	808	< 0.0001	9
Yolk b*	0.89	10.92	9	808	< 0.0001	10
Area	0.94	5.74	9	808	< 0.0001	11
Yolk color fan	0.94	5.73	9	808	< 0.0001	12
Eggshell strength	0.96	3.74	9	808	0.0001	13
Visual defects	0.96	3.44	9	808	0.0004	14
Eggshell thickness	0.96	3.38	9	808	0.0004	15
Yolk a*	0.97	2.54	9	808	0.0071	16
Yolk L*	0.98	2.18	9	808	0.0216	17
Albumen pH	0.98	1.66	9	808	0.0958	18
Yolk pH	0.99	1.18	9	808	0.3036	19

**Figure 2:**
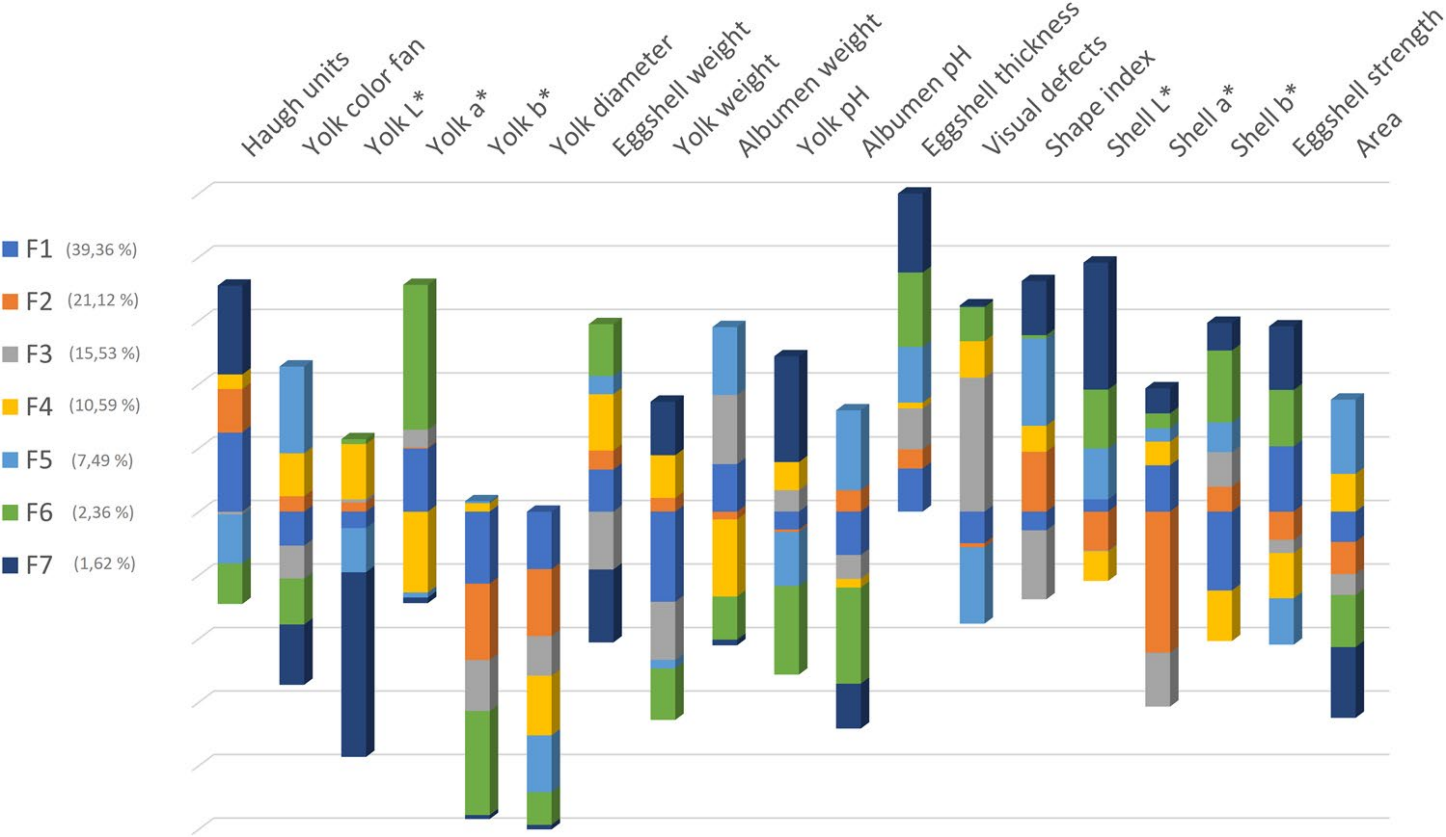
Discriminant loadings for external and internal quality-related traits determining the relative weight of each trait on each canonical discriminant function.

**Figure 3:**
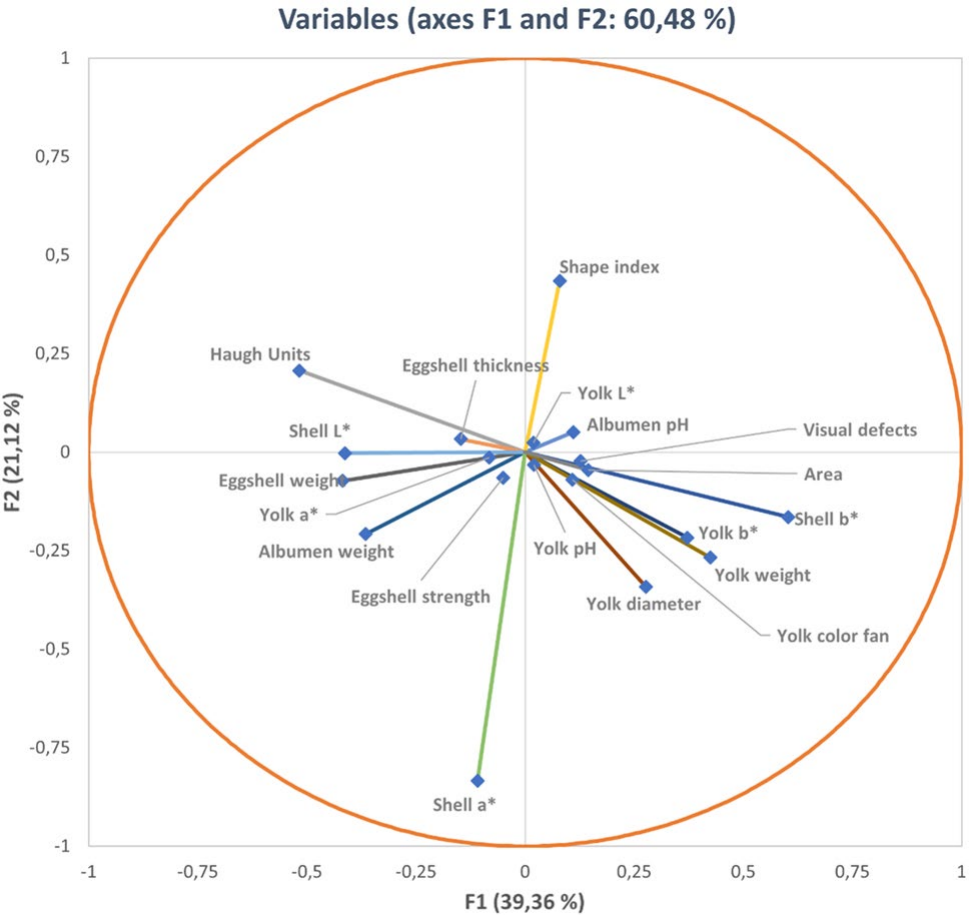
Vector plot for discriminant loadings for egg quality-related traits.

**Figure 4:**
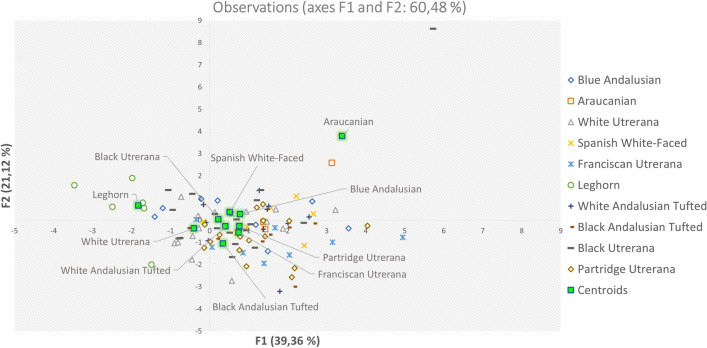
Territorial map depicting the eggs considered in the canonical discriminant analysis sorted across laying hen genotypes.

Standardized discriminant coefficients measure the relative weight of each egg quality trait across the discriminant functions (Figs. 2 and 3). Out of the seven significant discriminant functions (Table 2), only the two most relevant functions were used to build a standardized discriminant coefficient biplot, capturing the highest fraction of variance (Fig. 3). In this regard, those variables whose vector extends further apart from the origin most relevantly contributed to the first (F1) and second (F2) discriminant functions. Figure 4 suggests clear differentiation across eggs laid by the hens belonging to the different genotypes considered in the analyses. The relative position of centroids was determined through the substitution of the mean value for observations in each term of the first two discriminant functions (F1 and F2). The larger the distance between centroids, the better the predictive power of the canonical discriminant function in classifying observations.

**Figure 5:**
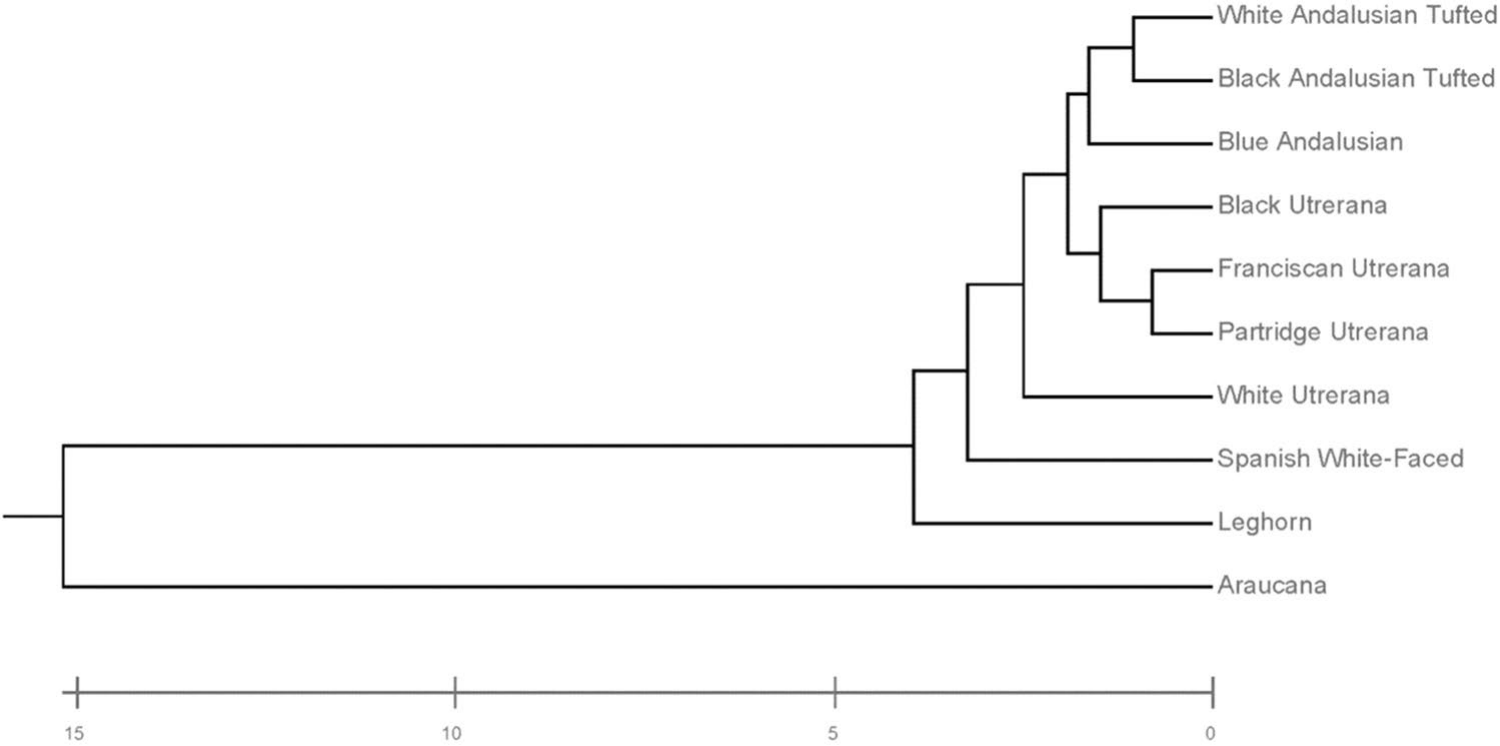
Cladogram constructed from Mahalanobis’s distances between laying hen genotypes.

Additionally, to evaluate the proximity between hen genotype clusters, Mahalanobis distances were represented (Fig. 5). Araucana hens were those most distantly located with respect to the rest of hen genotypes, with Andalusian Tufted black and white varieties’ eggs clustering together and further away from them than the rest of eggs. A certain connection is evidenced between black tufted, blue Andalusian and black Utrerana eggs. Nevertheless, a central Utrerana egg cluster revealed a closer relative relationship between black Utrerana and Franciscan and Partridge variety eggs. White Utrerana eggs were closely related to eggs laid by the rest of the Utrerana varieties, but a certain close connection was also reported with White-faced and Leghorn’s eggs.

The underlying basis for these classification patterns was found after the evaluation of the Data Mining CHAID Decision Tree obtained from the Chi-square dissimilarity matrix. In these regards, Chi squared bases branch and node distribution suggested eggs significantly (P < 0.05) differed, thus, were classified in five subgroups depending on their values of shell b* (≤ 1.49; 1.49–2.09; 2.09–2.91; 2.91–7.51; ≥ 7.51). Leghorn eggs predominate when shell b* values are lower than 2.09. Eggs with values of ≤ 1.49 and 2.91–7.51 for shell b* were classified by values of shape index; eggs with values of 2.09–2.91 for shell b* were classified depending on the yolk weight; and eggs with values of ≥ 7.51 for shell b* were classified according to egg weight. At the same time, eggs with values of 2.09–2.91 for shell b* and a yolk weighing more than 19.03 g were classified by area. This last distinction allowed the definition of mostly the eggs of the genotypes Blue Andalusian, White Andalusian Tufted and Franciscan Utrerana from those produced by Partridge Utrerana. Another subgroup was defined by eggs with values of ≥ 7.51 for shell b* and total egg weight of > 60.04 g. In this case, the shape index was quite important and separated black Andalusian tufted eggs (44.40% of eggs that showed values of > 7.51 for shell b*, > 60.40 g of total egg weight and ≤ 73.06 for shape index proceeded from black Andalusian tufted).

### Discriminant analysis and CHAID tree reliability: cross-validation

Supplementary Tables S2 and S3 report the results obtained in the classification and leave-one-out cross-validation. A Press′s Q value of 1939.49 (N = 819; n = 460; K = 10) was obtained. Therefore, it can be considered that predictions were significantly better than chance at 95%^16^. Afterwards, ten-fold cross validation reported similar resubstitution and cross-validation error rate estimates of 0.623 and 0.665, for which the standard error was 0.17 and 0.16, respectively, which determined that the CHAID tree built after the data in the study had reach the optimal depth. For these reasons, the robustness of the results obtained and the validity of the conclusions drawn from them can be supported.

## Discussion

The genetic diversity of farmed and domestic animals and the status of different genotypes can be understood following the Sustainable Development Goal 2.5.2. (Proportion of local breeds, classified as being at risk of extinction) and the classification of the risk status of animal populations provided by FAO guidelines^17,18^. Considering this classification, the risk status of the different local genotypes used in this research can be understood. While Utrerana breed status is endangered-maintained and Blue Andalusian breed status is critical-maintained, the risk status of Spanish White-Faced and Andalusian Tufted breeds is unknown since there are no breeder associations that are responsible for the management and control of the few existing populations^19^. In addition to this, a significant sample of the individuals of all located and registered breeders of each breed is kept in the conservation center of local breeds located in the Agropecuary Provincial Center of Diputación of Córdoba (Spain) where the experiment took place. Thus, the animal sample used in the present research and the variability of the egg quality traits given in Supplementary Table S1 is at the appropriate level to use the statistical analyzes and the conclusions offered in this study are statistically significant.

High correlations between major diameter and minor diameter with shape index can be explained by the fact that mathematical expression for shape index calculation (retained in the analyses) comprises the aforementioned parameters. On the other hand, egg weight, which was also deemed redundant and hence discarded, can be calculated from the sum of yolk, albumen, and eggshell weights. These findings are supported by previous research^15^, in which the same redundancies were detected. Additionally, it has been reported that it is necessary to verify the different relationships between the explanatory variables. In this way, selecting independent variables, instead of equations, produces that the variables do not overlap when deciding the factors that determine the efficiency of the predictive models, and therefore, optimizes the results obtained^20^.

The pH-related traits showed the lowest nonsignificant discriminating power between different groups of eggs (Table 3). The low contribution of pH-related traits to discriminating function may be derived from the low variability in egg pH found. pH can be taken as a measure of egg freshness: over time, there is a loss of CO_2_ and H_2_O inside the egg, accompanied by detrimental effects on egg quality, such as a decrease in flavor and albumen viscosity^21–23^. Although egg pH has been reported to be conditioned by the hen strain^24^, in the present research, pH measurements were taken within 24 h of oviposition. Therefore, these results may suggest that egg shell life may be affected by hen strains but at later stages; hence, the eggs in the present study could be considered fresh enough to avoid large variations in pH values between different breeds and varieties of hens. This explains the low variability in pH values across groups. Furthermore, considering that egg albumen and yolk pH values are correlated with embryo development^25^, and in light of the potential existence of differences at later stages, additional studies considering the evolution of pH along the storage time of eggs must be developed to determine breeds with higher egg shell life and to reinforce breeding strategies through conservation programs for endangered breeds for which egg shell life could be more easily compromised^6,26^.

High values of Wilks’ lambda and low values for F yolk color, measured by the L*a*b* color space and the yolk color fan systems, also suggested the limited discriminating potential of these traits. In this regard, while L* measures the degree of lightness, a* and b* parameters measure chromaticity: redness-greenness and yellowness-blueness, respectively. Photometric determination by spectrophotometer has been reported to be more precise than the yolk color fan^27^, with a similar discriminating power being reported for both parameters in the current study. Values for yolk b* were the most determinant yolk color-related parameter in the classification of eggs from different genotypes. These results are supported by Dvořák et al.^27^, who not only reported a significant negative correlation with other quality-related traits, such as total egg weight (r = − 0.919) and white weight (r = − 0.918) but also reported a broader distribution for absolute frequency values of the yolk b* (from 22.00 to 47.99) parameter when compared with yolk L* and yolk a*. Additionally, there was a strong mutual relation between yolk coloration parameters L* and b* (r = 0.927); hence, the deposition of yellow pigment in egg yolk could be presumed to be affected by the current metabolic capability of the hen, which has been reported to be a source for variability across breeds on which adaptability to the environment often relies^28^. Parallelly, the lowest discriminant relevance of a* parameter may be supported by the Dvořák et al.^27^, who elicited a* parameter to define red color spectrum component, with increasing egg yolk weight values being linked to decreased proportion of orange color which is preferred by consumers but, which however, has been reported to be independent from cholesterol concentration, thus egg internal quality^29^.

The variables area, eggshell strength and eggshell thickness were ranked 11th, 13th and 15th, respectively. However, eggshell weight was the best positioned shell-quality-related trait in the first half of the ranking. Previous studies have reported that egg weight values are not directly proportionally related to eggshell resistance^30^. The concentrations of Mg, Na and K in eggshell may be responsible for eggshell strength. High concentrations of these micronutrients in eggshell translate into increased egg fragility and softness^31^.

Leghorn eggshells have been reported to have greater concentrations of these micronutrients than local breeds^7^. For instance, Iqbal et al.^32^ observed that eggshell weight and eggshell thickness were positively correlated and significantly conditioned by egg size. Consequently, multicollinearity problems may derive from the strong relationship between eggshell thickness and weight, the reason why eggshell thickness may have been penalized (values of 0.964 for Wilks’ Lambda and 3.381 for F).

Visual defect parameters were shown to have low discriminating power. Blood and meat spots produce defects in the yolk and albumen of eggs, which cause rejection by egg consumers^9,33^. The rupture of an ovarian follicle at a different position from the stigma during ovulation and synthesis of different egg components could produce these visual defects^34^. The chromaticity of yolk can be altered by the presence of these spots, which could lead to high correlations between visual defects, yolk a*, and yolk b*^15^.

Yolk size-related parameters showed high discriminating power (6th and 8th positions for yolk weight and yolk diameter in the rank). These findings support the fact that hen genotype causes significant differences in the percentage of yolk. Several authors concluded that native breeds lay smaller eggs with a higher percentage of yolk than commercial hybrid strains^6,35,36^. The greater contribution of commercial lines of laying hens to the annual number of eggs and egg weight is produced at a more energetically efficient cost, laying eggs with a larger amount of albumen and therefore water^37^.

Albumen represents approximately 57–71% of the egg weight^38–40^. For this, albumen weight ranked first between the weight-related traits in the test for equality of group means. Leghorn eggs have been shown to have the heaviest eggshell (Supplementary Table S1). Hybrid strains have been subjected to high selective pressures in terms of eggshell quality due to their commercial and transport purposes^30^. However, in previous research, local genotypes have been shown to have stronger and stiffer eggshells than Leghorn’s genotypes, although these genotypes may present a lower eggshell weight than Leghorn’s genotypes^7^.

Haugh units ranked at the fifth position in the tests of equality of group means. It is used as an indicator of albumen quality. Haugh unit values are conditioned by storage conditions and time of storage^41^ but have also been reported to remarkably depend on hen genotype. When Haugh units are compared with yolk and white pH values, the differences may suggest that even if egg shell life has been reported to strongly vary across hen strains, variability occurs at later stages (with some breeds showing longer shell life periods than others), with reduced variability being found immediately after laying.

The results obtained in the present study are in accordance with previous research^24,42^, since they reported high values for Haugh units in selected lines of laying hens in comparison with native breeds. However, the percentage of albumen is directly correlated with albumen height^43^. Hence, the fact that commercial hybrid strains had a high percentage of albumen could provide a certain advantage to these genotypes in terms of more desirable Haugh unit values.

The shape index allows us to classify eggs as round eggs (shape index > 76), standard eggs (shape index = 72–76), and sharp eggs (shape index < 72)^44^. The high discriminating power reported by the trait shows great variability across the eggs of different genotypes used in the present study. While Araucana was reported to have round eggs (shape index = 76.84), white Utrerana eggs showed sharp shapes (shape index = 71.32).

Chromaticity parameters of eggshell reported the highest discriminating power. Although most of the genotypes used in the present study laid white-shelled eggs, the Araucana breed is distinguished by the laying of green–blue eggs^45^. Thus, shell a* and shell b* occupied the first positions in the rank in the test of equality of group means. Chromaticity parameters were responsible for Araucana breed clustering in a different group (Fig. 5). Nevertheless, even if the rest of the genotypes used in the study laid white-shelled eggs, shell b* allowed the classification of different breeds and varieties (Supplementary Fig. S1). For instance, the Leghorn breed showed values for shell b* close to 0. It has been suggested that high values for shell L* cause a decrease in values for shell b*^46^. In this context, according to Aygun^47^, if the eggshell L* value decreases (eggshell darkness increases), the Haugh unit value also decreases, but the shell strength increases. Hence, reporting the L* value on egg cartons could serve as a trace of differential breed quality marks.

Figure 5 reports the clear diversification of breeds depending on internal and external egg quality traits. In this regard, Araucana’s egg group differed from the rest of the Mediterranean and hybrid lines. Araucana geographic isolation may not only have promoted genetic and phenotypic distancing of this breed from the rest but also caused clear differentiation of its product^48,49^.

The separation of Spanish White-Faced and White Utrerana eggs in different clusters from the rest of autochthonous genotypes and their approach, in terms of egg quality to the commercial hybrid line, suggests that breeders could have crossed individuals with Leghorn hen, in an attempt to decrease consanguinity in Spanish White-Faced and White Utrerana, which account for the smallest number of animals and face a high endangerment risk. Nevertheless, the diversification of Leghorn eggs differed from the rest of native Spanish breeds’ eggs, suggesting that the aforementioned native breeds could constitute an alternative to eggs from other breeds that have traditionally been sold in the market^50^.

Similarities between egg quality-related traits of Partridge and Franciscan Utrerana were expected, since both varieties showed a higher proportion of yolk than the rest of the genotypes. On the other hand, Supplementary Fig. S1 suggests that Blue Andalusian eggs have, at the same time, similar characteristics to Black Utrerana and the two varieties of Andalusian Tufted breed. Eighty-one percent of eggs with values of > 7.51 for shell b*, > 60.04 g for egg weight and > 73.06 for shape index were laid by Blue Andalusian, Black Utrerana or Andalusian Tufted genotypes. Among the varieties of Utrerana hens, the black variety shares a high morphological resemblance with individuals of black plumage from the blue Andalusian breed^51^. Therefore, phenotypic similarities between these two genotypes, both morphological and productive, may indicate reminiscences of hybridization.

The closeness in the territorial map between eggs from the two varieties of Andalusian Tufted breed suggests a lack of reproductive management and crossbreeding between both varieties due to the low availability of animals belonging to the breed and the endangered situation the breed is facing. In addition, the absence of official recognition and a breeding program of certain local breeds can lead to a deterioration of the phenotypic and genotypic identity of their individuals^52^.

The present study develops a tool that allows efficient classification of eggs from 10 different genotypes based on quality-related traits as suggested by Press′s Q value exceeding 6.63, which denotes classification rate is at least 25% higher than that obtained by chance. This evidences, certain variables, such as shell a*, shell b*, albumen weight, shape index and Haugh units, play an important role in the determination of the external and internal quality of eggs. Indeed, a total of 91.18 and 61.90% of eggs of Leghorn and Araucana eggs, respectively, were correctly classified. However, 15.58% of Partridge Utrerana eggs were classified as Franciscan Utrerana eggs, and 20.48% of Black Andalusian Tufted eggs were classified as White Andalusian Tufted eggs. Furthermore, resubstitution error rate and the cross-validated error rate quotient was close to 1, as cross-validation risk did not significantly exceeded the risk of the minimum cross-validation risk tree plus one standard error, thus optimal tree depth was successfully attained.

Conclusively, the combination of discriminant canonical analysis and data mining CHAID decision trees methods is validated as an efficient tool to sort eggs from different genotypes considering quality egg traits. This tool enables the detection of hybridization trades or of the occurrence of mixing across breeds along their history. Certain external characteristics, such as chromaticity of eggshell and egg shape index, are easily measurable without the need to break the eggshell, and provide us with a large amount of information that allows us to correctly classify eggs from different genotypes. Among the different internal quality-related traits, albumen characteristics, such as Haugh units and albumen weight, play a pivotal role in the determination of differences across genotypes. Great differential egg quality features are reported when native breeds in Spain are compared to commercial hybrid lines or other foreign native breeds, such as the Araucana hen. These results complement those from genomic analyses as the latter concluded that some native varieties (white and black varieties) may still display evidence of a certain degree of hybridization with both commercial strains but also with other native breeds sharing the same area (Leghorn and Spanish Withe-Faced or White Utrerana hens and Black Utrerana and Andalusian Blue). In this regard, the similar proportions of the different parts of the egg (albumen, yolk, and shell) found in the Franciscan and Partridge varieties of the Utrerana breed may be a source of confusion for egg classification, while the egg of other genotypes, such as those of the Andalusian Tufted breed showed low product differentiation.

## Methods

### Institutional animal care and use committee statement

The study was conducted in accordance with the Declaration of Helsinki, the Royal Decree-national law 113/2013, of February 1, and the Directive 2010/63/EU of the European Parliament and of the Council of September 22. This study is out of the scope of evaluation of the Ethics Review Board of the University of Córdoba since it does not fall under legislation for the protection of animals used for scientific purposes. All methods are reported in accordance with ARRIVE guidelines and permission was granted by the authority of Agropecuary Provincial Center of Diputación of Córdoba (Spain) where the experiment took place.

### Layer flock and environmental conditions

The experiment took place at the Agropecuary Provincial Center of Diputación of Córdoba in southern Spain (37°54′50.9″N–4°42′40.4″W) for 1 year (from February 2019 to February 2020). The eggs used in the present study were obtained from a flock of layers comprising animals belonging to different breeds distributed as described in Table 4. Half of the individuals of each local breed were pullets (24 weeks old) and half hens (70 weeks old). However, in the Leghorn Lohmann LSL-Classic lineage flock, all animals used for the study were pullets (24 weeks old). The selection of the sampled individuals was performed considering the age when the different used genotypes reach 50% of laying (egg production during a laying cycle). Contextually, the typical production cycle in commercial layers (Leghorn hens among others) lasts about 72 weeks^53^. However, this cycle may extend until 156 weeks in around a third of the Utrerana population^54^.

**Table 4:** Number of individuals (N) used in each studied breed and variety.

Breed and variety	n	Age
White Utrerana	15	Half of the individuals of each local breed were pullets (24 weeks old) and half hens (70 weeks old)
Franciscan Utrerana	15	
Black Utrerana	15	
Partridge Utrerana	15	
Blue Andalusian	10	
Spanish White-Faced	8	
White Andalusian Tufted	8	
Black Andalusian Tufted	8	
Araucana	4	
Leghorn Lohmann LSL-Classic lineage	10	Pullets (24 weeks old)

Hens themselves did not participate in any experiment described in the present study but were the source of the eggs from whom eggs were collected. The birds from which the eggs were collected were placed in pens, with a stocking density of 1 animal per m^2^, and were fed the same commercial feed (chemical composition: 15.20% crude protein, 4.60% crude fats and oil, 3.20% crude fiber, 14.00% crude ash, 4.10% calcium, 0.66% phosphorus, 0.19% sodium, 0.31% methionine, 0.72% lysine). Water and feed were provided ad libitum.

### Work sample

A total of 819 eggs were sampled for egg quality measurement. Eggs were laid during a complete laying cycle. Table 5 shows the classification of eggs depending on the laying hen genotype. The same information registration protocol was performed individually for all the eggs of the sample.

**Table 5:** Number of observations (n) sampled for egg quality measurement in each studied breed and variety.

Breed and variety	n
White Utrerana	98
Franciscan Utrerana	109
Black Utrerana	95
Partridge Utrerana	77
Blue Andalusian	45
Spanish White-Faced	47
White Andalusian Tufted	73
Black Andalusian Tufted	84
Araucana	21
Leghorn Lohmann LSL-Classic lineage	170
**Total**	819

### Measurements of external and internal quality-related traits

External quality-related traits were measured following noninvasive methods, that is, without breaking the eggshell. The following external egg quality trait measures were evaluated: major and minor diameters of eggs; egg weight; eggshell color lightness, redness-greenness and yellowness-blueness coordinates (shell L*, shell a*, and shell b*), and shape index.

On the other hand, when the egg had to be broken to be evaluated, the scored internal egg quality-related traits were as follows: eggshell weight; eggshell thickness; eggshell resistance, composed of eggshell strength and area under the force–displacement curve (area); albumen height; Haugh units; albumen weight; albumen pH; yolk pH; yolk color fan; yolk lightness, redness, and yellowness variables (yolk L*, yolk a*, and yolk b*); yolk diameter; yolk weight; and the presence or absence of visual defects in yolk and/or albumen. Haugh units and shape index (Table 6) were calculated following the premises established by Eisen et al.^55^ and Anderson et al.^56^. The colour of the shell was determined using a portable spectrophotometer (CM 700d, Konica Minolta Holdings Inc., Tokyo, Japan), and the results were expressed using the International Commission on Illumination (CIE) L*a*b* system color profile as described in González Ariza et al.^6^.

**Table 6:** Mathematical description of the egg quality-related indices.

Trait	Mathematical expression	
Shape index	$SI = (\emptyset M/\emptyset m) \times 100$	Where SI: shape index; $\emptyset$M: major diameter; $\emptyset$m: minor diameter
Haugh units	$HU = 100 \times {\text{log}}(h - 1.7w^{0.37} + 7.6)$	Where HU: Haugh units; h: albumen height (mm); w: egg weight (g)

**Figure 6:**
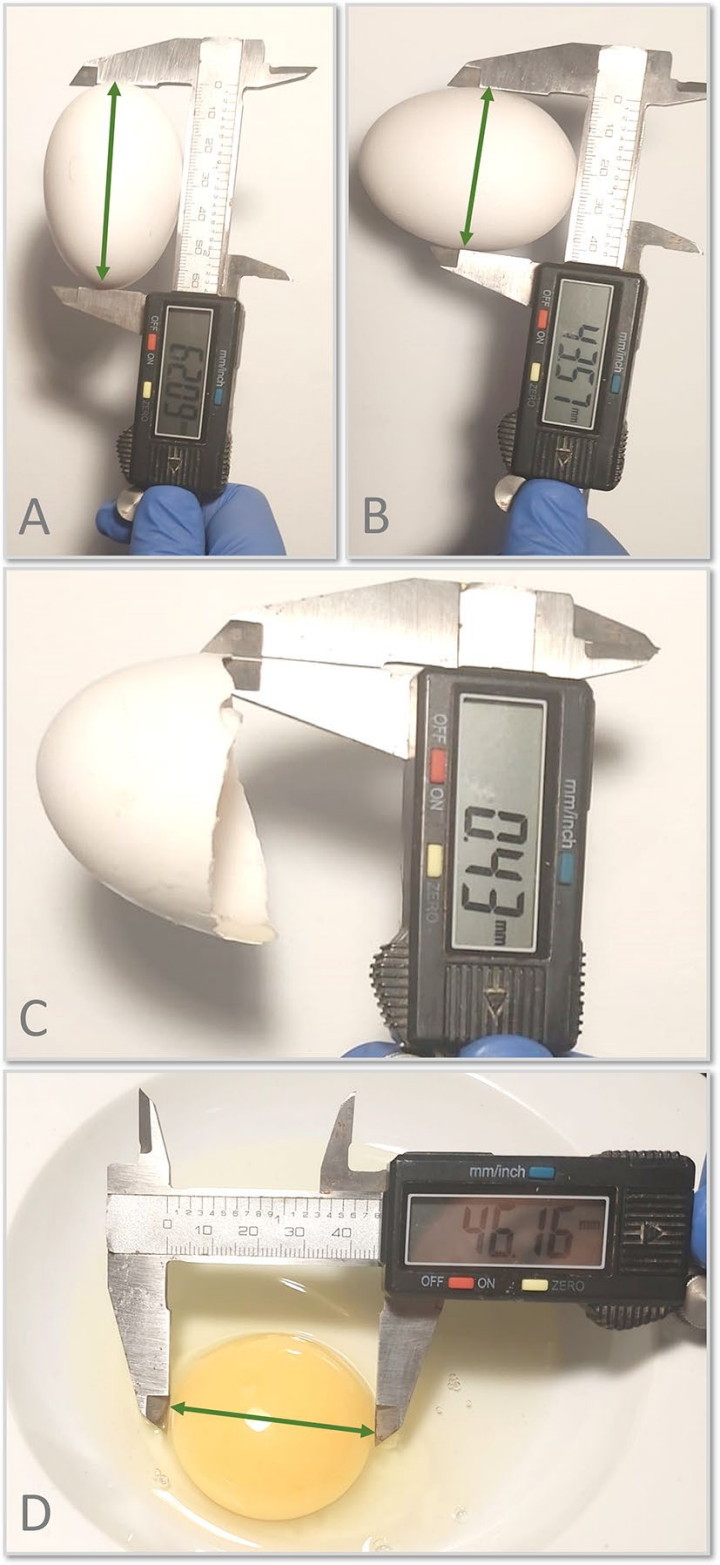
Scheme of the external and internal egg quality data collection procedure. (**A**) Major diameter; (**B**) Minor diameter; (**C**) Eggshell thickness; (**D**) Yolk diameter.

Figure 6 depicts the detailed procedure for measurement collection of the following variables: major and minor diameters, eggshell thickness, and yolk diameter. For this, a Vernier scale (Electro DH M 60.205, Barcelona, Spain) was used. The eggshell thickness was computed as the mean of three measurements taken at the central part of the eggshell. The egg quality evaluation was measured within 24 h after oviposition every 15 days for one year. The room temperature was 22 ± 1 °C at the time of the egg quality evaluation. Further information regarding the data collection protocol used can be found in González Ariza et al.^15^.

### Canonical discriminant analysis

Canonical discriminant analyses (CDAs) were performed to design a tool that enables the classification of eggs while determining whether linear combinations of measures of internal and external egg quality-related traits describe within- and between-population group clustering patterns. The explanatory variables used for the present analyses were major diameter, minor diameter, egg weight, shell L*, shell a*, shell b*, shape index, eggshell weight, eggshell thickness, eggshell strength, area, albumen height, Haugh units, albumen weight, albumen pH, yolk pH, yolk color fan, yolk L*, yolk a*, yolk b*, yolk diameter, yolk weight, and visual defects. The genotype of the laying hen was considered the clustering criterion.

Canonical relationships with traits were plotted to depict the group differences into an easily interpretable territorial map. Regularized forward stepwise multinomial logistic regression algorithms were used to perform the variable selection. Priors were regularized according to the group sizes calculated using the prior probability of commercial software (SPSS Version 26.0 for Windows, SPSS, Inc., Chicago, IL) instead of considering them the same to avoid groups with different sample sizes affecting the quality of the classification^20^.

The same sample size contexts as those used in this study across groups have been reported to be robust. In this regard, some authors have reported a minimum sample size of at least 20 observations for every 4 or 5 predictors, and the maximum number of independent variables should be n-2, where n is the sample size, to palliate possible distortion effects^20,57^.

Consequently, the present study used a 4 or 5 times higher ratio between observations and independent variables than those described above, which renders discriminant approaches efficient. Multicollinearity analysis was run to ensure independence and a strong linear relationship across predictors. Variables chosen by the forward or backward stepwise selection methods were the same. Finally, the progressive forward selection method was performed since it requires less time than the backward selection method.

The discriminant routine of the Classify package of SPSS version 26.0 software and the canonical discriminant analysis routine of the Analyzing Data package of XLSTAT software (Addinsoft Pearson Edition 2014, Addinsoft, Paris, France) were used to perform canonical discriminant analysis.

### Multicollinearity preliminary testing

The multicollinearity assumption must be tested before running a discriminant canonical analysis to ensure that redundancies in the variables considered do not overinflate the variance explanatory potential. This is to discard variables which explain the same fraction of variability in data as others considered within the analyses as well, but which may not be able to explain certain additional fractions which other variables do. For example, egg weight may be explained by eggshell, yolk and albumen weights, but still the latter may be able to represent a rather precise fraction of the differences across observations. Hence, preserving all in the analyses may determine a redundant explanation (inflation) of such variability. Multicollinearity is a data condition which represents a high degree of linear intercorrelation between two or more explanatory variables. Whereas correlation is the linear relationship between just two variables, multicollinearity can exist between two variables or between one variable and the linear combination of the others. Multicollinearity also represents a lack of orthogonality among variables, this means changes in one of them do not imply changes in the rest. Different methods can be used to detect multicollinearity. Among them, variance inflation factor (VIF) and tolerance^58^, measures the ratio of variance in a regression model with multiple attributes divided by the variance of a model with only one attribute^59^. Multicollinearity occurs when *k* vectors lie in a subspace of dimension less than *k*. Multicollinearity can explain a data-poor condition, which frequently is found in observational studies in which the researchers do not interfere with the study. Thus, many investigators often confuse multicollinearity with correlation. Therefore, correlation is considered a special case of multicollinearity. A high correlation implies multicollinearity, but not the other way around. There may be multicollinearity between the explanatory variables, but still not a high correlation between pairs of these variables^60^. A recommended VIF value of 4 was used in the study^61^. VIF was computed according to the following formula as a subroutine of the Canonical Discriminant Analysis routine of the Analyzing Data package of XLSTAT software (Addinsoft Pearson Edition 2014, Addinsoft, Paris, France):

$$VIF = 1/(1 - R^{2} ),$$

where R^2^ is the coefficient of determination of the regression equation.

### Canonical correlation dimension determination

The maximum number of canonical correlations between two sets of variables is the number of variables in the smaller set. The first canonical correlation usually explains most of the relationships between different sets. In any case, attention should be given to all canonical correlations, despite reporting of only the first dimension being common in previous research^62^. When canonical correlation values are 0.30 or higher, they correspond to approximately 10% of the variance explained.

### Canonical discriminant analysis efficiency

Wilks’ lambda test evaluates which variables may significantly contribute to the discriminant function. When Wilks’ lambda approximates 0, the contribution of that variable to the discriminant function increases. χ^2^ tests the Wilks’ Lambda significance. If significance is below 0.05, the function can be concluded to explain the group adscription well^63^.

### Canonical discriminant analysis model reliability

Pillai’s trace criterion, as the only acceptable test to be used in cases of unequal sample sizes, was used to test the assumption of equal covariance matrices in the discriminant function analysis^64^. Pillai’s trace criterion was computed as a subroutine of the Canonical Discriminant Analysis routine of the Analyzing Data package of XLSTAT software (Addinsoft Pearson Edition 2014, Addinsoft, Paris, France). A significance of ≤ 0.05 is indicative of the set of predictors considered in the discriminant model being statistically significant. Pillai's trace criterion is argued to be the most robust statistic for general protection against departures from the multivariate residuals’ normality and homogeneity of variance. The higher the observed value for Pillai’s trace is, the stronger the evidence that the set of predictors has a statistically significant effect on the values of the response variable. That is, the Pillai trace criterion shows potential linear differences in the combined internal and external egg quality traits across hen genotype clustering groups^65^.

### Canonical coefficients and loading interpretation and spatial representation

When CDA is implemented, a preliminary principal component analysis is used to reduce the overall variables into a few meaningful variables that contributed most to variations between eggs from different genotypes. The use of the CDA determined the percentage assignment of eggs within its own group. Variables with a discriminant loading of ≥ |0.40| were considered substantive, indicating substantive discriminating variables. By the use of the stepwise procedure technique, nonsignificant variables were prevented from entering the function. Coefficients with large absolute values correspond to variables with greater discriminating ability. Data were standardized following procedures reported by Manly and Alberto^66^. Then, squared Mahalanobis distances and principal component analysis were computed using the following formula:

$$D_{ij}^{2} = (\overline{\Upsilon }_{i} - \overline{\Upsilon }_{j} ) COV^{ - 1} (\overline{\Upsilon }_{i} - \overline{\Upsilon }_{j} ) ,$$

where $D_{ij}^{2}$: distance between population i and j; COV^−1^: inverse of the covariance matrix of measured variable x; $\overline{\Upsilon }_{i}$ and $\overline{\Upsilon }_{j}$: means of variable x in the ith and jth populations, respectively.

The squared Mahalanobis distance matrix was converted into a Euclidean distance matrix, and a dendrogram was built using the underweighted pair-group method arithmetic averages (UPGMA; Rovira i Virgili University, Tarragona, Spain) and the Phylogeny procedure of MEGA X 10.0.5 (Institute of Molecular Evolutionary Genetics, The Pennsylvania State University, State College, PA, USA).

### Discriminant function reliability: cross-validation

Afterwards, to determine the probability that an egg of an unknown background belongs to a particular classification group^67^, the hit ratio parameter was computed. For this, the relative distance of the problem observation to the centroid of its closest group was used. The hit ratio is the percentage of correctly classified eggs that is correctly ascribed to the hen genotype that originally laid them. The leave-one-out cross-validation procedure is used as a form of significance to consider if the discriminant functions can be validated.

Press′s Q statistic can support these results, since it can be used to compare the discriminating power of the cross-validated function, as follows:

$$Press^{\prime}s\; Q = \frac{{[n - (n^{\prime}K)]^{2} }}{n(K - 1)},$$

where n: number of observations in the sample; n′: number of observations correctly classified; K: number of groups.

The value of Press′s Q statistic must be compared with the critical value of 6.63 for χ^2^ with a degree of freedom at a significance of 0.01. When Press′s Q exceeds the critical value of χ^2^ = 6.63, the cross-validated classification rate is at least 25% higher than that obtained by chance and classification accuracy levels enough can be considered achieved.

### Data mining CHAID decision tree

The Chi-squared automatic interaction detection (CHAID) decision tree (DT) data mining method was used for classification, prediction, interpretation, and discretely categorized data manipulation. The CHAID-based algorithm decision support tool includes a root node, branches, and leaf nodes. For each internal node to be built around an egg quality trait (input variables), a Chi-square test significance split criterion (P < 0.05) must be fulfilled (prepruning). According to Breiman et al.^68^, pruning (either pre or post) processes must be implemented to prevent trees from presenting a large number of branches and to prevent them from failing to pursue branches that can add significantly to the overall fit. After computing a tree exhaustively depicting the significant relationship across independent variables detected, nodes that do not contribute to the overall prediction are discarded. Furthermore, CHAID adds an element of penalization as an indirect cost derived from model complexity. In this regard, Bonferroni inequality was used to significantly adjust for significance levels. Breiman’s method resembles forward stepwise regression with a cutting back on the final number of steps using chi squared tests instead of *F*-to-enter-based tests. Each branch represents an outcome of the test (in a number of two or more), and each leaf node (or terminal node) represents a category level of the target variable (hen genotype). The top most node in a tree is the root node. The decisions are made at each node, and each record of data continues through the tree along a path until the record reaches a leaf or terminal node of the tree^69^.

### Data mining CHAID decision tree reliability: cross-validation

Afterwards, cross validation was performed to validate the set of predictors considered to measure the differences between the prediction error for a tree applied to a new sample and a training sample. Cross-validation of the decision tree was performed using the ‘complexity parameter’ and cross-validated error to estimate how accurately the model generalizes for unseen data, i.e.; how well it performs/predicts. Ten-fold cross-validation was used to validate the CHAID decision tree and ensure that the set of predictors considered significantly explains the differences across breed groups^70^. This means to determine whether the shortest tree efficiently and repeatably collects the highest number of significant relationships. All sample records of the training sample and the study data were used to perform the ten-fold cross-validation. Cross-validation was performed by comparing the existing differences between the prediction error for a tree applied to a new sample (resubstitution/replacement error rate) and a training sample (cross-validation error rate). The cross-validation error rate (risk) is an averaging of the risks across the 10 test samples (folds, new samples) and determines data prediction discriminant model accuracy. The process is repeated for each fold, and an estimate of the error across folds is estimated. The tree that produced the lowest cross-validation error rate and, therefore, presented the best fit was selected. By contrast, the resubstitution error rate is the proportion of original misclassified observations by various subsets of the original tree and decreases as the depth of the tree increases. While the tree reporting the lowest resubstitution rate will be biased, large trees add random variation in the predictions as they overfit outliers. As a consequence, optimal tree depth is determined on the shallowest tree whose cross-validation risk does not exceed the risk of the minimum cross-validation risk tree plus one standard error. This can be ensured when resubstitution error rate and the cross-validated error rate are similar, hence, their quotient is close to 1.

## Supplementary Information

Supplementary Figure S1.

Supplementary Table S1.

Supplementary Table S2.

Supplementary Table S3.

Supplementary Table S4.

## Data Availability

The datasets generated and/or analyzed during the current study are available from the corresponding author on reasonable request.
