# Supplementary material for: Data mining-based discriminant analysis as a tool for the study of egg quality in native hen breeds
Source: Sci Rep. 2022 Sep 23;12:15873. doi: 10.1038/s41598-022-20111-z (PMC9508079; doi:10.1038/s41598-022-20111-z)
Supplement: Supplementary file 1 — Supplementary Figure S1. [file 41598_2022_20111_MOESM1_ESM.docx]

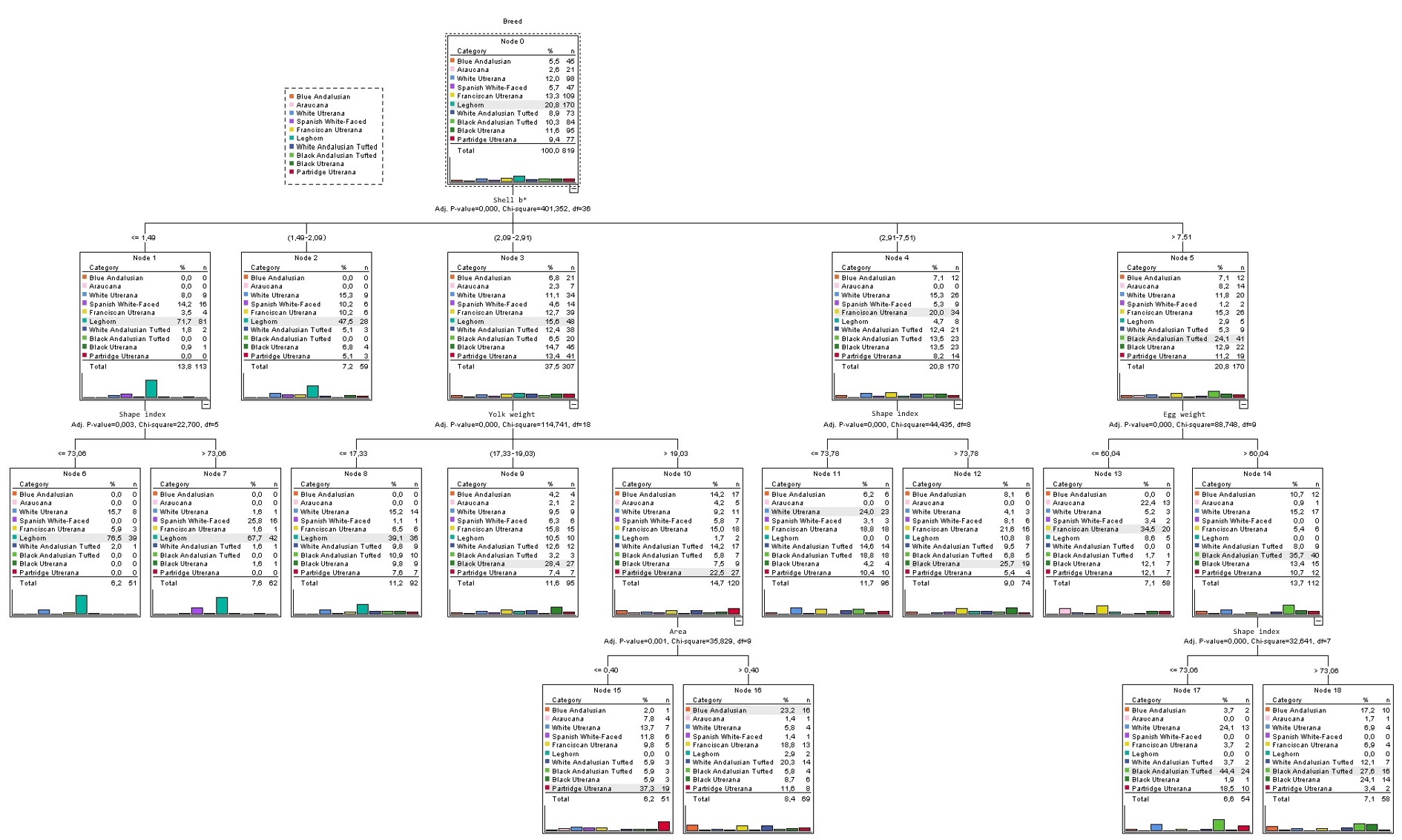


**Supplementary Figure S1.** Data Mining CHAID Decision Tree obtained from the chi-square dissimilarity matrix among egg quality traits across hen breed genotypes.
