# Supplementary material for: Data mining-based discriminant analysis as a tool for the study of egg quality in native hen breeds
Source: Sci Rep. 2022 Sep 23;12:15873. doi: 10.1038/s41598-022-20111-z (PMC9508079; doi:10.1038/s41598-022-20111-z)
Supplement: Supplementary file 2 — Supplementary Table S1. [file 41598_2022_20111_MOESM2_ESM.docx]

**Supplementary Table S1.** Summary of descriptive statistics for egg quality-related traits in each studied group.

|  |  |  | Albumen height | Haugh Units | Yolk Color fan | Yolk L* | Yolk a* | Yolk b* | Yolk diameter | Eggshell weight | Yolk weight | Albumen weight | Yolk pH | Albumen pH | Eggshell thickness | Egg weight | Major diameter | Minor diameter | Shape index | Shell L* | Shell a* | Shell b* | Eggshell strength | Area |
| --- | --- | --- | --- | --- | --- | --- | --- | --- | --- | --- | --- | --- | --- | --- | --- | --- | --- | --- | --- | --- | --- | --- | --- | --- |
| Andalusian Blue | Mean | | 7.59 | 84.78 | 12.79 | 50.76 | 8.16 | 37.24 | 43.98 | 8.67 | 19.94 | 34.64 | 6.24 | 8.52 | 0.38 | 64.82 | 58.89 | 44.46 | 75.60 | 85.98 | -0.35 | 7.86 | 4.32 | 0.43 |
|  | Std. Deviation | | 1.59 | 10.61 | 1.36 | 4.18 | 3.65 | 9.16 | 1.88 | 1.61 | 1.80 | 4.30 | 0.17 | 0.33 | 0.07 | 4.86 | 2.47 | 1.10 | 3.27 | 8.77 | 0.94 | 4.11 | 1.06 | 0.11 |
|  | Minimum | | 3.51 | 49.50 | 11.00 | 42.41 | 2.36 | 25.87 | 40.15 | 5.74 | 17.20 | 25.39 | 5.95 | 7.94 | 0.24 | 54.84 | 54.49 | 42.01 | 69.90 | 61.37 | -1.17 | 2.59 | 1.24 | 0.10 |
|  | Maximum | | 10.88 | 101.98 | 16.00 | 60.63 | 17.26 | 55.82 | 47.99 | 13.57 | 23.60 | 42.79 | 6.62 | 9.67 | 0.60 | 76.40 | 64.28 | 46.49 | 82.53 | 93.27 | 2.78 | 16.27 | 5.85 | 0.61 |
|  | Percentiles | 25% | 6.49 | 79.25 | 12.00 | 47.91 | 4.88 | 29.19 | 42.72 | 7.45 | 18.56 | 32.26 | 6.12 | 8.24 | 0.34 | 61.45 | 56.88 | 43.92 | 73.27 | 85.53 | -0.88 | 3.97 | 3.62 | 0.40 |
|  |  | 50% | 7.67 | 85.65 | 13.00 | 50.36 | 7.90 | 35.72 | 43.80 | 8.57 | 19.47 | 34.77 | 6.22 | 8.60 | 0.38 | 65.66 | 59.03 | 44.56 | 74.92 | 88.70 | -0.68 | 6.59 | 4.64 | 0.45 |
|  |  | 75% | 9.12 | 93.44 | 14.00 | 54.20 | 10.31 | 46.05 | 45.01 | 9.43 | 21.42 | 38.13 | 6.35 | 8.71 | 0.42 | 67.88 | 60.67 | 45.30 | 78.47 | 91.28 | -0.27 | 10.30 | 5.00 | 0.50 |
| Araucanian | Mean | | 6.37 | 80.74 | 11.83 | 52.32 | 7.88 | 32.98 | 42.23 | 6.37 | 18.63 | 27.55 | 6.22 | 8.58 | 0.36 | 54.24 | 54.65 | 41.97 | 76.84 | 81.47 | -4.45 | 12.20 | 3.13 | 0.36 |
|  | Std. Deviation | | 1.19 | 8.50 | 0.99 | 3.32 | 3.10 | 6.26 | 1.60 | 0.50 | 1.10 | 4.10 | 0.14 | 0.32 | 0.09 | 4.20 | 1.94 | 1.05 | 1.81 | 4.44 | 2.46 | 4.80 | 0.91 | 0.06 |
|  | Minimum | | 3.87 | 57.66 | 10.00 | 45.32 | 1.90 | 24.00 | 40.36 | 5.01 | 17.21 | 21.73 | 6.05 | 7.90 | 0.22 | 47.73 | 51.63 | 40.55 | 73.96 | 73.68 | -7.79 | 2.14 | 1.90 | 0.25 |
|  | Maximum | | 8.27 | 93.29 | 13.00 | 56.57 | 11.36 | 47.64 | 45.64 | 7.03 | 20.61 | 35.14 | 6.70 | 9.02 | 0.55 | 61.34 | 57.69 | 43.98 | 80.59 | 90.56 | 0.49 | 20.46 | 4.85 | 0.50 |
|  | Percentiles | 25% | 5.41 | 75.27 | 11.00 | 50.01 | 5.46 | 28.58 | 40.66 | 6.09 | 17.66 | 24.22 | 6.14 | 8.37 | 0.30 | 50.72 | 52.82 | 41.06 | 75.67 | 77.88 | -6.56 | 9.89 | 2.50 | 0.32 |
|  |  | 50% | 6.36 | 82.86 | 12.00 | 52.13 | 9.24 | 32.75 | 41.86 | 6.46 | 18.53 | 26.18 | 6.20 | 8.64 | 0.34 | 53.21 | 54.45 | 41.98 | 76.55 | 81.32 | -4.69 | 11.22 | 2.80 | 0.37 |
|  |  | 75% | 7.35 | 86.38 | 13.00 | 54.96 | 10.09 | 35.28 | 43.63 | 6.73 | 19.62 | 30.74 | 6.28 | 8.84 | 0.42 | 58.44 | 56.64 | 42.88 | 78.15 | 83.83 | -3.28 | 17.51 | 3.86 | 0.40 |
| Black Utrerana | Mean | | 7.45 | 84.78 | 12.85 | 50.76 | 7.70 | 36.01 | 42.60 | 8.38 | 18.50 | 34.61 | 6.27 | 8.45 | 0.40 | 62.88 | 58.22 | 43.94 | 75.56 | 88.25 | -0.17 | 6.89 | 4.14 | 0.45 |
|  | Std. Deviation | | 1.44 | 8.90 | 1.29 | 4.44 | 3.81 | 8.99 | 2.23 | 1.35 | 1.88 | 5.43 | 0.16 | 0.36 | 0.07 | 7.42 | 2.92 | 1.70 | 2.84 | 6.30 | 1.40 | 4.15 | 1.27 | 0.12 |
|  | Minimum | | 3.50 | 57.83 | 8.00 | 42.01 | -1.66 | 19.87 | 36.46 | 5.13 | 11.58 | 20.20 | 5.95 | 7.70 | 0.25 | 45.41 | 50.79 | 39.23 | 68.22 | 51.57 | -8.24 | 0.72 | 1.50 | 0.10 |
|  | Maximum | | 10.68 | 102.34 | 15.00 | 62.47 | 14.79 | 65.70 | 47.85 | 11.99 | 23.30 | 47.50 | 6.88 | 9.13 | 0.62 | 81.43 | 66.34 | 48.15 | 81.81 | 94.31 | 2.60 | 18.00 | 6.60 | 0.71 |
|  | Percentiles | 25% | 6.73 | 80.78 | 12.00 | 47.66 | 5.17 | 29.51 | 41.20 | 7.35 | 17.43 | 30.86 | 6.17 | 8.17 | 0.36 | 57.31 | 56.22 | 42.78 | 73.87 | 87.34 | -0.66 | 3.23 | 3.29 | 0.39 |
|  |  | 50% | 7.43 | 85.77 | 13.00 | 49.99 | 7.83 | 34.12 | 42.61 | 8.49 | 18.47 | 34.23 | 6.25 | 8.43 | 0.39 | 61.84 | 58.23 | 43.93 | 75.59 | 89.06 | -0.36 | 6.51 | 4.18 | 0.46 |
|  |  | 75% | 8.37 | 89.61 | 14.00 | 52.86 | 10.68 | 42.54 | 44.41 | 9.39 | 19.36 | 38.78 | 6.36 | 8.79 | 0.46 | 68.32 | 60.34 | 45.14 | 77.17 | 91.46 | 0.42 | 8.74 | 5.31 | 0.51 |
| Franciscan Utrerana | Mean | | 6.89 | 82.17 | 12.25 | 52.98 | 8.37 | 36.33 | 43.62 | 8.15 | 19.10 | 30.96 | 6.22 | 8.43 | 0.38 | 59.13 | 58.34 | 42.59 | 73.09 | 87.84 | 0.20 | 6.95 | 3.98 | 0.40 |
|  | Std. Deviation | | 1.49 | 9.71 | 1.59 | 4.05 | 3.71 | 7.91 | 2.17 | 1.05 | 1.95 | 4.15 | 0.15 | 0.31 | 0.07 | 5.71 | 2.38 | 1.42 | 2.95 | 6.53 | 1.24 | 4.56 | 1.21 | 0.13 |
|  | Minimum | | 2.53 | 48.79 | 8.00 | 43.90 | -1.64 | 23.28 | 38.52 | 4.80 | 14.91 | 20.02 | 5.75 | 7.15 | 0.20 | 38.48 | 47.71 | 38.20 | 66.56 | 54.74 | -1.30 | 0.63 | 1.20 | 0.06 |
|  | Maximum | | 11.44 | 104.50 | 16.00 | 62.94 | 16.32 | 60.58 | 50.83 | 11.46 | 25.30 | 43.31 | 6.70 | 9.06 | 0.57 | 73.83 | 65.91 | 45.80 | 82.75 | 95.08 | 5.72 | 24.48 | 6.39 | 0.80 |
|  | Percentiles | 25% | 6.27 | 77.71 | 11.00 | 50.10 | 6.07 | 30.36 | 42.19 | 7.46 | 17.87 | 28.35 | 6.12 | 8.24 | 0.33 | 13.61 | 6.06 | 3.43 | 6.63 | 16.88 | 8.28 | 18.32 | 2.95 | 0.25 |
|  |  | 50% | 6.93 | 83.53 | 12.00 | 52.91 | 8.27 | 35.33 | 43.59 | 7.96 | 18.96 | 30.66 | 6.22 | 8.46 | 0.37 | 36.02 | 15.55 | 8.92 | 13.58 | 42.74 | 10.84 | 17.28 | 5.10 | 0.61 |
|  |  | 75% | 7.67 | 87.71 | 13.00 | 55.72 | 10.32 | 40.16 | 45.03 | 8.77 | 20.05 | 33.52 | 6.31 | 8.67 | 0.42 | 62.66 | 59.95 | 43.60 | 74.72 | 91.87 | 0.70 | 10.81 | 4.82 | 0.48 |
| Leghorn | Mean | | 9.13 | 94.50 | 11.95 | 51.74 | 8.73 | 27.89 | 42.05 | 8.89 | 16.83 | 35.43 | 6.24 | 8.42 | 0.40 | 62.68 | 58.82 | 43.61 | 74.27 | 92.30 | -0.65 | 1.66 | 3.99 | 0.36 |
|  | Std. Deviation | | 1.07 | 5.50 | 1.35 | 4.45 | 2.93 | 6.49 | 2.81 | 1.26 | 2.81 | 4.57 | 0.28 | 0.35 | 0.06 | 7.19 | 3.17 | 1.70 | 3.55 | 2.96 | 0.36 | 1.93 | 1.32 | 0.15 |
|  | Minimum | | 6.46 | 78.43 | 7.00 | 33.60 | 1.17 | 14.77 | 33.08 | 6.33 | 9.86 | 23.79 | 5.11 | 7.05 | 0.19 | 40.23 | 47.26 | 37.57 | 65.04 | 69.17 | -1.43 | -0.60 | 1.00 | 0.02 |
|  | Maximum | | 11.56 | 104.92 | 15.00 | 63.18 | 18.02 | 56.29 | 48.33 | 11.94 | 30.78 | 50.92 | 6.90 | 9.10 | 0.57 | 93.38 | 69.06 | 50.04 | 89.57 | 95.43 | 2.67 | 12.67 | 7.37 | 0.80 |
|  | Percentiles | 25% | 8.45 | 91.50 | 11.00 | 49.00 | 6.71 | 24.40 | 40.28 | 7.94 | 15.05 | 32.89 | 6.07 | 8.17 | 0.36 | 57.31 | 56.22 | 42.78 | 73.87 | 87.34 | -0.66 | 3.23 | 3.29 | 0.39 |
|  |  | 50% | 9.22 | 95.60 | 12.00 | 52.09 | 8.65 | 26.98 | 42.14 | 8.63 | 16.89 | 35.23 | 6.21 | 8.48 | 0.40 | 62.27 | 58.53 | 43.63 | 74.35 | 92.81 | -0.66 | 1.16 | 4.07 | 0.40 |
|  |  | 75% | 9.89 | 98.05 | 13.00 | 55.20 | 10.61 | 29.91 | 43.94 | 9.97 | 18.62 | 38.22 | 6.37 | 8.69 | 0.44 | 66.85 | 60.35 | 44.66 | 76.66 | 94.34 | -0.59 | 1.98 | 4.87 | 0.46 |
|  |  | |  |  |  |  |  |  |  |  |  |  |  |  |  |  |  |  |  |  |  |  |  |  |
|  |  | |  |  |  |  |  |  |  |  |  |  |  |  |  |  |  |  |  |  |  |  |  |  |
| Partridge Utrerarna | Mean | | 7.68 | 86.45 | 12.32 | 51.50 | 6.96 | 36.74 | 43.82 | 7.74 | 19.46 | 33.09 | 6.30 | 8.45 | 0.38 | 61.87 | 59.21 | 43.17 | 73.03 | 86.95 | -0.34 | 7.01 | 3.87 | 0.41 |
|  | Std. Deviation | | 1.40 | 8.69 | 1.18 | 3.91 | 3.44 | 10.22 | 2.30 | 1.05 | 2.53 | 4.31 | 0.18 | 0.30 | 0.07 | 6.48 | 2.96 | 1.54 | 3.43 | 8.04 | 0.72 | 3.98 | 0.82 | 0.08 |
|  | Minimum | | 4.15 | 57.16 | 8.00 | 43.20 | -0.97 | 18.90 | 38.21 | 4.84 | 14.02 | 20.00 | 6.00 | 7.88 | 0.14 | 44.63 | 52.73 | 39.85 | 59.95 | 55.60 | -1.50 | 1.53 | 2.21 | 0.20 |
|  | Maximum | | 11.64 | 107.72 | 15.00 | 60.46 | 16.36 | 62.62 | 48.10 | 10.31 | 23.89 | 44.15 | 6.85 | 9.02 | 0.63 | 77.42 | 67.87 | 46.10 | 81.45 | 94.62 | 1.38 | 20.81 | 6.11 | 0.60 |
|  | Percentiles | 25% | 6.85 | 82.52 | 12.00 | 49.52 | 5.20 | 28.51 | 42.22 | 6.94 | 17.35 | 30.12 | 6.18 | 8.20 | 0.34 | 57.04 | 56.58 | 42.11 | 70.94 | 86.28 | -0.85 | 3.90 | 3.39 | 0.35 |
|  |  | 50% | 7.60 | 86.81 | 12.00 | 51.81 | 7.38 | 36.10 | 43.88 | 7.68 | 19.48 | 33.54 | 6.29 | 8.44 | 0.38 | 62.04 | 59.19 | 43.30 | 72.92 | 89.58 | -0.54 | 6.95 | 3.86 | 0.41 |
|  |  | 75% | 8.54 | 92.18 | 13.00 | 53.40 | 8.68 | 45.44 | 45.81 | 8.40 | 21.49 | 35.42 | 6.36 | 8.73 | 0.41 | 65.89 | 61.24 | 44.33 | 75.27 | 92.05 | -0.11 | 9.93 | 4.43 | 0.47 |
| Black And. Tufted | Mean | | 7.63 | 85.34 | 12.14 | 50.54 | 9.06 | 34.32 | 44.57 | 8.13 | 19.42 | 36.19 | 6.25 | 8.46 | 0.38 | 64.68 | 60.42 | 43.91 | 72.77 | 88.21 | 0.86 | 8.44 | 3.97 | 0.38 |
|  | Std. Deviation | | 1.47 | 9.21 | 1.11 | 4.78 | 2.74 | 9.77 | 2.11 | 1.31 | 2.70 | 4.21 | 0.25 | 0.36 | 0.08 | 6.47 | 2.93 | 1.12 | 2.39 | 3.20 | 1.41 | 3.31 | 1.11 | 0.13 |
|  | Minimum | | 4.00 | 54.06 | 10.00 | 36.95 | 1.31 | 18.93 | 39.25 | 5.61 | 15.65 | 26.28 | 5.58 | 7.27 | 0.19 | 54.59 | 55.22 | 41.54 | 67.09 | 81.99 | -1.59 | 2.47 | 1.00 | 0.04 |
|  | Maximum | | 10.59 | 100.43 | 15.00 | 63.56 | 13.51 | 58.54 | 49.62 | 12.55 | 36.30 | 46.26 | 6.93 | 9.11 | 0.65 | 84.26 | 67.70 | 47.15 | 78.00 | 94.40 | 4.19 | 15.13 | 6.11 | 0.60 |
|  | Percentiles | 25% | 6.71 | 81.77 | 11.00 | 47.52 | 7.35 | 27.93 | 43.12 | 7.17 | 17.77 | 32.99 | 6.14 | 8.26 | 0.33 | 60.28 | 58.29 | 43.12 | 70.96 | 85.59 | -0.22 | 5.90 | 3.43 | 0.35 |
|  |  | 50% | 7.66 | 86.40 | 12.00 | 50.26 | 9.00 | 31.97 | 44.42 | 7.94 | 19.36 | 35.73 | 6.22 | 8.48 | 0.37 | 0.37 | 0.37 | 0.37 | 0.37 | 0.41 | 0.41 | 0.41 | 0.37 | 0.37 |
|  |  | 75% | 8.69 | 91.42 | 13.00 | 53.14 | 11.41 | 37.76 | 46.26 | 8.70 | 20.69 | 38.59 | 6.31 | 8.75 | 0.43 | 68.14 | 61.93 | 44.50 | 74.29 | 90.32 | 1.99 | 11.05 | 4.77 | 0.45 |
| White And. Tufted | Mean | | 7.29 | 83.81 | 12.09 | 53.11 | 8.36 | 35.93 | 44.19 | 7.98 | 19.05 | 34.72 | 6.24 | 8.55 | 0.37 | 62.62 | 59.33 | 43.38 | 73.23 | 90.31 | -0.31 | 4.82 | 3.69 | 0.41 |
|  | Std. Deviation | | 1.59 | 9.56 | 1.30 | 4.15 | 3.53 | 7.56 | 2.25 | 1.39 | 2.31 | 4.96 | 0.19 | 0.32 | 0.07 | 7.91 | 3.09 | 1.76 | 3.29 | 2.84 | 0.77 | 3.38 | 1.20 | 0.14 |
|  | Minimum | | 3.81 | 58.16 | 7.00 | 44.79 | -0.66 | 24.40 | 39.43 | 5.81 | 13.84 | 24.57 | 6.00 | 7.84 | 0.19 | 54.59 | 55.22 | 41.54 | 67.09 | 81.99 | -1.59 | 2.47 | 1.00 | 0.04 |
|  | Maximum | | 11.72 | 103.69 | 15.00 | 61.69 | 14.10 | 59.06 | 49.39 | 11.92 | 23.96 | 47.57 | 6.83 | 9.00 | 0.52 | 0.52 | 0.52 | 0.52 | 0.52 | 0.57 | 0.57 | 0.57 | 0.52 | 0.52 |
|  | Percentiles | 25% | 6.18 | 77.79 | 11.50 | 49.22 | 5.20 | 30.69 | 42.32 | 6.77 | 17.64 | 30.56 | 6.11 | 8.27 | 0.32 | 57.42 | 56.98 | 42.44 | 70.93 | 88.24 | -0.63 | 2.71 | 2.76 | 0.35 |
|  |  | 50% | 7.26 | 85.14 | 12.00 | 53.41 | 9.08 | 34.06 | 44.59 | 7.80 | 19.02 | 34.74 | 6.17 | 8.65 | 0.37 | 36.02 | 15.55 | 8.92 | 13.58 | 42.74 | 10.84 | 17.28 | 5.10 | 0.61 |
|  |  | 75% | 8.24 | 90.51 | 13.00 | 56.25 | 11.17 | 39.51 | 46.07 | 8.85 | 20.47 | 38.93 | 6.31 | 8.83 | 0.41 | 67.40 | 61.33 | 44.49 | 75.71 | 92.86 | -0.20 | 6.55 | 4.41 | 0.51 |
| White Utrerana | Mean  Std. Deviation | | 7.85 | 86.89 | 11.89 | 52.06 | 7.42 | 32.85 | 42.68 | 8.00 | 18.01 | 35.93 | 6.27 | 8.41 | 0.37 | 63.46 | 60.66 | 43.20 | 71.32 | 88.74 | -0.18 | 5.54 | 3.46 | 0.33 |
|  |  |  | 1.47 | 9.65 | 1.34 | 4.49 | 3.41 | 9.34 | 2.17 | 1.02 | 2.32 | 3.69 | 0.19 | 0.32 | 0.06 | 5.45 | 2.82 | 1.15 | 2.90 | 7.19 | 0.75 | 4.44 | 1.12 | 0.12 |
|  | Minimum | | 4.07 | 53.87 | 7.00 | 41.28 | -1.42 | 19.28 | 35.19 | 5.58 | 13.43 | 26.91 | 5.90 | 7.65 | 0.24 | 48.09 | 53.02 | 39.36 | 62.93 | 53.45 | -1.76 | -0.11 | 0.70 | 0.04 |
|  | Maximum | | 10.83 | 102.32 | 15.00 | 64.47 | 15.50 | 63.90 | 48.17 | 10.36 | 31.23 | 46.59 | 6.85 | 9.06 | 0.54 | 76.37 | 68.60 | 46.40 | 78.47 | 99.48 | 2.62 | 19.46 | 6.46 | 0.60 |
|  | Percentiles | 25% | 7.08 | 84.21 | 11.00 | 49.14 | 5.47 | 26.42 | 41.41 | 7.21 | 16.71 | 33.36 | 6.13 | 8.17 | 0.33 | 60.14 | 58.60 | 42.57 | 69.20 | 86.89 | -0.64 | 2.05 | 2.71 | 0.28 |
|  |  | 50% | 8.06 | 89.66 | 12.00 | 52.31 | 7.35 | 30.74 | 42.85 | 8.00 | 17.82 | 36.06 | 6.24 | 8.39 | 0.37 | 63.01 | 60.58 | 43.37 | 71.42 | 89.89 | -0.36 | 3.98 | 3.48 | 0.35 |
|  |  | 75% | 9.01 | 93.88 | 13.00 | 54.48 | 9.45 | 35.80 | 43.97 | 8.77 | 19.26 | 38.74 | 6.36 | 8.68 | 0.41 | 66.92 | 62.50 | 44.02 | 73.37 | 93.36 | 0.22 | 9.09 | 4.37 | 0.42 |
| Spanish White-Faced | Mean  Std. Deviation | | 7.52 | 86.76 | 11.78 | 52.06 | 7.60 | 37.58 | 42.95 | 7.86 | 18.45 | 27.09 | 6.26 | 8.50 | 0.36 | 54.67 | 55.56 | 42.30 | 76.16 | 91.29 | -0.59 | 2.80 | 4.00 | 0.38 |
|  |  |  | 2.03 | 12.49 | 0.84 | 4.42 | 2.02 | 10.06 | 2.06 | 0.90 | 2.44 | 3.35 | 0.14 | 0.28 | 0.06 | 4.69 | 1.75 | 1.24 | 2.12 | 2.64 | 0.31 | 2.87 | 0.89 | 0.08 |
|  | Minimum | | 3.13 | 52.52 | 10.00 | 40.35 | 1.93 | 21.53 | 38.47 | 6.07 | 14.21 | 19.02 | 6.01 | 7.99 | 0.16 | 44.90 | 51.89 | 39.47 | 72.08 | 82.38 | -1.69 | 0.27 | 1.30 | 0.10 |
|  | Maximum | | 12.79 | 112.16 | 14.00 | 60.46 | 10.96 | 67.30 | 47.78 | 10.64 | 30.60 | 33.46 | 6.66 | 9.39 | 0.50 | 63.51 | 59.03 | 44.81 | 81.42 | 94.51 | -0.04 | 12.21 | 5.56 | 0.51 |
|  | Percentiles | 25% | 6.03 | 78.26 | 11.00 | 50.86 | 6.57 | 31.44 | 41.13 | 7.28 | 17.32 | 24.95 | 6.16 | 8.24 | 0.32 | 50.69 | 54.34 | 41.47 | 74.70 | 90.46 | -0.73 | 0.84 | 3.55 | 0.35 |
|  |  | 50% | 7.29 | 87.26 | 12.00 | 51.88 | 7.57 | 35.23 | 43.43 | 7.87 | 18.53 | 27.06 | 6.26 | 8.47 | 0.35 | 54.71 | 55.43 | 42.40 | 76.32 | 91.90 | -0.55 | 1.56 | 4.16 | 0.39 |
|  |  | 75% | 8.75 | 96.13 | 12.00 | 54.93 | 9.35 | 44.52 | 44.39 | 8.44 | 19.30 | 29.48 | 6.33 | 8.73 | 0.39 | 58.53 | 56.70 | 43.16 | 77.33 | 93.14 | -0.43 | 4.00 | 4.54 | 0.42 |

Albumen height, yolk diameter, eggshell thickness, major diameter, and minor diameter measurements are expressed in millimeters; eggshell weight, yolk weight, albumen weight, and egg weight measurements are expressed in grams; eggshell strength measurements are expressed in kilograms; are measurements are expressed in kilograms/seconds.
