# Supplementary material for: Data mining-based discriminant analysis as a tool for the study of egg quality in native hen breeds
Source: Sci Rep. 2022 Sep 23;12:15873. doi: 10.1038/s41598-022-20111-z (PMC9508079; doi:10.1038/s41598-022-20111-z)
Supplement: Supplementary file 3 — Supplementary Table S2. [file 41598_2022_20111_MOESM3_ESM.docx]

| **Statistics/Parameters** | **Tolerance (1 – R^2^)** | **VIF** |
| --- | --- | --- |
| Major diameter | 0.00 | 245.72 |
| Shape index | 0.01 | 183.38 |
| Minor diameter | 0.01 | 122.58 |
| Albumen height | 0.02 | 41.82 |
| Haugh units | 0.03 | 39.16 |
| Egg weight | 0.05 | 20.47 |
| Albumen weight | 0.19 | 5.14 |
| Yolk weight | 0.37 | 2.72 |
| Eggshell weight | 0.42 | 2.38 |
| Shell b* | 0.46 | 2.19 |
| Yolk diameter | 0.47 | 2.11 |
| Shell L* | 0.48 | 2.08 |
| Eggshell strength | 0.54 | 1.87 |
| Yolk color fan | 0.58 | 1.73 |
| Resistance area | 0.58 | 1.71 |
| Eggshell thickness | 0.68 | 1.47 |
| Yolk a* | 0.68 | 1.47 |
| Yolk L* | 0.74 | 1.35 |
| Yolk b* | 0.78 | 1.29 |
| Albumen pH | 0.80 | 1.25 |
| Shell a* | 0.82 | 1.22 |
| Yolk pH | 0.88 | 1.14 |
| Visual defects | 0.95 | 1.05 |

**Supplementary Table S2.** Complete procedure of progressive forward selection method for multicollinearity analysis of quality-related traits of eggs. Interpretation thumb rule: VIF = 1 (Not correlated); 1 < VIF < 4 (Moderately correlated); VIF ≥ 4 (Highly correlated).

**Table S2a.** First step.

**Table S2b.** Second step.

| **Statistics/Parameters** | **Tolerance (1 – R^2^)** | **VIF** |
| --- | --- | --- |
| Albumen height | 0.02 | 41.76 |
| Haugh units | 0.03 | 39.10 |
| Egg weight | 0.05 | 20.47 |
| Minor diameter | 0.06 | 15.73 |
| Albumen weight | 0.20 | 5.05 |
| Shape index | 0.22 | 4.51 |
| Yolk weight | 0.37 | 2.70 |
| Eggshell weight | 0.42 | 2.39 |
| Shell b* | 0.46 | 2.19 |
| Yolk diameter | 0.48 | 2.10 |
| Shell L* | 0.48 | 2.07 |
| Eggshell strength | 0.54 | 1.87 |
| Yolk color fan | 0.58 | 1.73 |
| Resistance area | 0.58 | 1.71 |
| Eggshell thickness | 0.68 | 1.47 |
| Yolk a* | 0.68 | 1.46 |
| Yolk L* | 0.74 | 1.35 |
| Yolk b* | 0.78 | 1.29 |
| Albumen pH | 0.80 | 1.25 |
| Shell a* | 0.82 | 1.22 |
| Yolk pH | 0.88 | 1.14 |
| Visual defects | 0.96 | 1.05 |

| **Statistics/Parameters** | **Tolerance (1 – R^2^)** | **VIF** |
| --- | --- | --- |
| Egg weight | 0.05 | 19.67 |
| Minor diameter | 0.06 | 15.69 |
| Albumen weight | 0.20 | 5.04 |
| Shape index | 0.22 | 4.51 |
| Yolk weight | 0.37 | 2.69 |
| Eggshell weight | 0.44 | 2.30 |
| Shell b* | 0.46 | 2.18 |
| Yolk diameter | 0.48 | 2.10 |
| Shell L* | 0.48 | 2.07 |
| Eggshell strength | 0.54 | 1.86 |
| Yolk color fan | 0.58 | 1.72 |
| Resistance area | 0.59 | 1.71 |
| Eggshell thickness | 0.68 | 1.47 |
| Yolk a* | 0.69 | 1.46 |
| Yolk L* | 0.74 | 1.35 |
| Yolk b* | 0.78 | 1.28 |
| Haugh units | 0.80 | 1.25 |
| Albumen pH | 0.80 | 1.25 |
| Shell a* | 0.82 | 1.23 |
| Yolk pH | 0.88 | 1.13 |
| Visual defects | 0.96 | 1.04 |

**Table S2c.** Third step.

| **Statistics/Parameters** | **Tolerance (1 – R^2^)** | **VIF** |
| --- | --- | --- |
| Minor diameter | 0.16 | 6.42 |
| Albumen weight | 0.23 | 4.29 |
| Yolk weight | 0.38 | 2.61 |
| Shape index | 0.44 | 2.27 |
| Shell b* | 0.46 | 2.17 |
| Eggshell weight | 0.47 | 2.13 |
| Yolk diameter | 0.48 | 2.10 |
| Shell L* | 0.48 | 2.07 |
| Eggshell strength | 0.54 | 1.86 |
| Yolk color fan | 0.58 | 1.71 |
| Resistance area | 0.59 | 1.70 |
| Yolk a* | 0.69 | 1.45 |
| Eggshell thickness | 0.69 | 1.44 |
| Yolk L* | 0.74 | 1.34 |
| Yolk b* | 0.78 | 1.28 |
| Haugh units | 0.80 | 1.24 |
| Albumen pH | 0.80 | 1.24 |
| Shell a* | 0.82 | 1.21 |
| Yolk pH | 0.88 | 1.13 |
| Visual defects | 0.96 | 1.05 |

**Table S2d.** Fourth step.

| **Statistics/Parameters** | **Tolerance (1 – R^2^)** | **VIF** |
| --- | --- | --- |
| Yolk weight | 0.46 | 2.18 |
| Shell b* | 0.46 | 2.18 |
| Shell L* | 0.48 | 2.08 |
| Yolk diameter | 0.48 | 2.07 |
| Eggshell weight | 0.53 | 1.88 |
| Eggshell strength | 0.54 | 1.86 |
| Yolk color fan | 0.58 | 1.71 |
| Resistance area | 0.59 | 1.70 |
| Albumen weight | 0.67 | 1.50 |
| Yolk a* | 0.69 | 1.45 |
| Eggshell thickness | 0.69 | 1.45 |
| Yolk L* | 0.74 | 1.35 |
| Yolk b* | 0.78 | 1.28 |
| Albumen pH | 0.80 | 1.24 |
| Haugh units | 0.81 | 1.23 |
| Shell a* | 0.83 | 1.20 |
| Shape index | 0.86 | 1.16 |
| Yolk pH | 0.89 | 1.13 |
| Visual defects | 0.96 | 1.04 |

**Table S2e.** Last step.
