# Supplementary material for: Data mining-based discriminant analysis as a tool for the study of egg quality in native hen breeds
Source: Sci Rep. 2022 Sep 23;12:15873. doi: 10.1038/s41598-022-20111-z (PMC9508079; doi:10.1038/s41598-022-20111-z)
Supplement: Supplementary file 4 — Supplementary Table S3. [file 41598_2022_20111_MOESM4_ESM.docx]

| **from \ to** | **Blue Andalusian** | **Araucana** | **White Utrerana** | **Spanish White-Faced** | **Franciscan Utrerana** | **Leghorn** | **White Andalusian Tufted** | **Black Andalusian Tufted** | **Black Utrerana** | **Partridge Utrerana** | **Total** | **% correct** |
| --- | --- | --- | --- | --- | --- | --- | --- | --- | --- | --- | --- | --- |
| **Blue Andalusian** | 18 | 0 | 1 | 4 | 4 | 4 | 8 | 3 | 2 | 1 | 45 | 40.00 |
| **Araucana** | 0 | 13 | 0 | 2 | 1 | 0 | 2 | 1 | 0 | 2 | 21 | 61.90 |
| **White Utrerana** | 1 | 0 | 55 | 0 | 3 | 17 | 6 | 5 | 8 | 3 | 98 | 56.12 |
| **Spanish White-Faced** | 0 | 0 | 0 | 28 | 8 | 5 | 4 | 0 | 1 | 1 | 47 | 59.57 |
| **Franciscan Utrerana** | 3 | 0 | 5 | 6 | 53 | 8 | 13 | 8 | 6 | 7 | 109 | 48.62 |
| **Leghorn** | 2 | 0 | 2 | 1 | 4 | 155 | 2 | 0 | 4 | 0 | 170 | 91.18 |
| **White Andalusian Tufted** | 6 | 0 | 7 | 3 | 6 | 4 | 30 | 11 | 4 | 2 | 73 | 41.10 |
| **Black Andalusian Tufted** | 4 | 0 | 2 | 1 | 9 | 8 | 17 | 41 | 1 | 0 | 83 | 49.40 |
| **Black Utrerana** | 6 | 1 | 8 | 3 | 11 | 7 | 7 | 4 | 42 | 6 | 95 | 44.21 |
| **Partridge Utrerana** | 4 | 0 | 7 | 3 | 12 | 4 | 10 | 3 | 9 | 25 | 77 | 32.47 |
| **Total** | 44 | 14 | 87 | 51 | 111 | 212 | 99 | 76 | 77 | 47 | 818 | 56.23 |

**Supplementary Table S3.** Appropriately classified eggs according to the genotype of the laying hen.
