# Supplementary material for: Data mining-based discriminant analysis as a tool for the study of egg quality in native hen breeds
Source: Sci Rep. 2022 Sep 23;12:15873. doi: 10.1038/s41598-022-20111-z (PMC9508079; doi:10.1038/s41598-022-20111-z)
Supplement: Supplementary file 5 — Supplementary Table S4. [file 41598_2022_20111_MOESM5_ESM.docx]

| **from \ to** | **Blue Andalusian** | **Araucana** | **White Utrerana** | **Spanish White-Faced** | **Franciscan Utrerana** | **Leghorn** | **White Andalusian Tufted** | **Black Andalusian Tufted** | **Black Utrerana** | **Partridge Utrerana** | **Total** | **% correct** |
| --- | --- | --- | --- | --- | --- | --- | --- | --- | --- | --- | --- | --- |
| **Blue Andalusian** | 13 | 0 | 1 | 5 | 5 | 5 | 8 | 4 | 3 | 1 | 45 | 28.89 |
| **Araucana** | 0 | 13 | 0 | 2 | 1 | 0 | 2 | 1 | 0 | 2 | 21 | 61.90 |
| **White Utrerana** | 1 | 0 | 48 | 0 | 3 | 17 | 7 | 6 | 10 | 6 | 98 | 48.98 |
| **Spanish White-Faced** | 0 | 0 | 0 | 25 | 9 | 7 | 4 | 0 | 1 | 1 | 47 | 53.19 |
| **Franciscan Utrerana** | 4 | 0 | 5 | 7 | 42 | 8 | 15 | 11 | 8 | 9 | 109 | 38.53 |
| **Leghorn** | 2 | 0 | 3 | 1 | 4 | 152 | 4 | 0 | 4 | 0 | 170 | 89.41 |
| **White Andalusian Tufted** | 6 | 0 | 10 | 3 | 6 | 4 | 26 | 11 | 5 | 2 | 73 | 35.62 |
| **Black Andalusian Tufted** | 4 | 0 | 3 | 1 | 10 | 9 | 17 | 38 | 1 | 0 | 83 | 45.78 |
| **Black Utrerana** | 7 | 1 | 8 | 3 | 12 | 8 | 7 | 4 | 38 | 7 | 95 | 40.00 |
| **Partridge Utrerana** | 4 | 0 | 9 | 3 | 14 | 4 | 13 | 3 | 10 | 17 | 77 | 22.08 |
| **Total** | 41 | 14 | 87 | 50 | 106 | 214 | 103 | 78 | 80 | 45 | 818 | 50.37 |

**Supplementary Table S4.** Leave-one-out cross-validation of eggs according to the genotype of the laying hen.
